# High H_2_O-Assisted Proton Conduction in One Highly Stable Sr(II)-Organic Framework Constructed by Tetrazole-Based Imidazole Dicarboxylic Acid

**DOI:** 10.3390/molecules29112656

**Published:** 2024-06-04

**Authors:** Junyang Feng, Ying Li, Lixia Xie, Jinzhao Tong, Gang Li

**Affiliations:** 1 School of Pharmaceutical Engineering, Henan Technical Institute, Zhengzhou 450042, China; feng329426@sina.com (J.F.); yechenmo1989@126.com (Y.L.); 2 College of Science, Henan Agricultural University, Zhengzhou 450002, China; henauxlx@henau.edu.cn; 3 College of Chemistry, Zhengzhou University, Zhengzhou 450001, China; tongjch2023@lzu.edu.cn

**Keywords:** metal–organic framework, strontium(II), proton conduction, mechanism

## Abstract

Solid electrolyte materials with high structural stability and excellent proton conductivity (σ) have long been a popular and challenging research topic in the fuel cell field. This problem can be addressed because of the crystalline metal–organic frameworks’ (MOFs’) high structural stability, adjustable framework composition, and dense H-bonded networks. Herein, one highly stable Sr(II) MOF, {[Sr(H_2_tmidc)_2_(H_2_O)_3_]·4H_2_O}_n_ (**1**) (H_3_tmidc = 2-(1H-tetrazolium-1-methylene)-1H-imidazole-4,5-dicarboxylic acid) was successfully fabricated, which was structurally characterized by single-crystal X-ray diffraction and electrochemically examined by the AC impedance determination. The results demonstrated that the σ of the compound manifested a positive dependence on temperature and humidity, and the optimal proton conductivity is as high as 1.22 × 10^−2^ S/cm under 100 °C and 98% relative humidity, which is at the forefront of reported MOFs with ultrahigh σ. The analysis of the proton conduction mechanism reveals that numerous tetrazolium groups, carboxyl groups, coordination, and crystallization water molecules in the framework are responsible for the high efficiency of proton transport. This work offers a fresh perspective on how to create novel crystalline proton conductive materials.

## 1. Introduction

With the growth of the population and the advancement of science and technology, the problem of fossil energy scarcity and environmental pollution will arrive sooner than expected. As a result, all governments now prioritize the development of renewable clean energy. The proton exchange membrane fuel cell (PEMFC), a mature renewable energy consumption device, has been extensively explored [1,2,3]. The proton exchange membrane (PEM), as the “heart” of PEMPC, not only allows protons to pass through the membrane and links the circuit, but it also regulates the intensity of the reaction, allowing control over the battery’s power and life [4,5,6]. Nafion and its derivative membranes are currently the most common commercial PEMs, with a proton conductivity (σ) of up to 10^−2^ S/cm. Although the Nafion membrane has a large commercial market, its high cost, difficult preparation, and tight operating conditions make it unsuitable for most people’s needs. Its proton conduction mechanism is particularly challenging to characterize due to its amorphous form [7]. As a result, much effort has been directed into the creation of novel alternative proton-conductive materials.

In this context, metal–organic frameworks (MOFs) are ideal for proton-conductive materials because of their modified pores, regular pore topologies, and ease of crystallization. As a result, in recent years, they have received a great deal of attention [8,9,10,11,12,13,14,15,16]. Proton-conducting MOF research is currently in full swing. According to the organic ligand classification, relevant research includes MOFs produced with phosphonic acid [17], carboxylate [9], *N*-heterocyclic carboxylic acid [18], sulfonic acid ligands [10], and so on [19]. According to the metal salt classification, the primary research includes practically all metal elements in the periodic table, including the main group MOFs [20], transition metals [11], and lanthanide metals [21].

Organic ligands and their functional groups have long been recognized as important in forming proton-conductive MOFs. For instance, carboxylic acid groups carried by organic ligands, can not only coordinate with metal ions to constitute a stable MOF framework but can also act as proton sources and carriers, as well as participate in the construction of H-bonds within the framework to provide proton transport pathways [22,23,24,25,26,27,28,29]. Moreover, it has also been discovered that the electron absorption induction of *N*-heterocyclic groups such as imidazole and triazole increases the acidity of the carboxyl group, thereby improving the structural stability of MOFs, resulting in high water resistance and chemical inertia, laying the groundwork for future practical applications [18,30,31,32,33]. For example, in 2012, the Banerjee group used In(III) and Cd(II) metal salt to react with 5-triazoline phenylic acid (5-TIA) to generate two proton-conducting MOFs, In-5TIA and Cd-5TIA [34]. Under 301 K and 98% relative humidity (RH), their σ values were as high as 5.35 × 10^−5^ and 3.61 × 10^−3^ S/cm. Our research group has used substituted imidazole dicarboxylic acid ligands to assemble some structurally stable MOFs for proton conduction investigation. We have obtained some exciting findings, such as the optimal proton conductivity of up to 10^−3^ S/cm [35,36,37,38,39]. At the same time, free *N*-heterolytic small molecules can be employed as a proton source or carrier in a MOF’s framework, resulting in high-efficiency proton transmission [40,41,42,43]. However, these post-modified molecules are not anchored firmly enough in the MOF framework, resulting in leakage defects during use.

Given the above, we intend to introduce multiple nitrogen heterocyclic groups directly into organic carboxylic acid compounds via pre-design and then self-assemble with metal ions as multifunctional bridging ligands to prepare structurally stable MOFs containing both carboxylic and *N*-heterocyclic groups for proton conduction research.

Herein, the ligand of 2-(1H-tetrazolium-1-methylene)-1H-imidazole-4,5-dicarboxylic acid (H_3_tmidc) bearing imidazole and tetrazole and carboxylate units was selected to react with strontium chloride hexahydrate, obtaining a new Sr(II) MOF, {[Sr(H_2_tmidc)_2_(H_2_O)_3_]·4H_2_O}_n_ (**1**), which was characterized structurally by single-crystal X-ray diffraction. Consequently, its high thermal, H_2_O, and chemical stabilities were confirmed by thermogravimetric (TG) analysis and a powder X-ray diffraction (PXRD) test. Furthermore, the compound’s σ was investigated in a humid environment. The results revealed that its proton conductivity boosted with rising temperature or RH and reached the highest point at 100 °C and 98% RH, up to 1.22 × 10^−2^ S/cm, which is at the forefront of known high-performance proton-conductive MOFs. Finally, crystal structure analysis and estimated activation energy values illustrate the framework’s proton transport mechanism.

## 2. Results and Discussion

### 2.1. Crystal Structure

**Figure 1:**
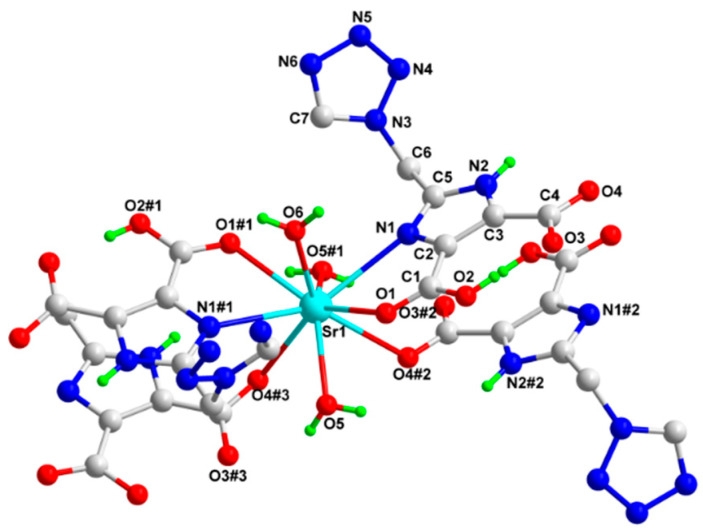
The coordination surroundings of Sr(II) atom (Blue: N; Red: O; Green: H; Gray: C; ultramarine: Sr. Symmetry modes: #1:x + 2, y, −z + 3/2; #2: −x + 2, −y + 3, −z + 2).

MOF **1** crystallized in the monoclinic crystal system, C2/c space group. Each asymmetric unit constitutes 0.5 Sr(II) ions, 1 H_2_tmidc^−^ anion, 1.5 coordinated H_2_O units, and 2 solvent H_2_O units. As illustrated in Figure 1, each Sr(II) ion was coordinated by two N1 and N1#1 atoms from the H_2_tmidc^−^ anion, four O1, O1#1, O4#2, and O4#3 atoms from the H_2_tmidc^−^ anion, and three water molecules (O5, O5#1, and O6) to form a twisted SrN_2_O_7_ coordination environment.

The dihedral angle between the imidazole and the tetrazole planes is 73.345(6)°, and the dihedral angles between the imidazole unit and the two carboxyl planes are 4.48(7)° and 9.68(9)°, respectively. Sr-N distances are 2.8083(12) and 2.8084(12) Å, respectively, while Sr-O distances vary from 2.545(2) to 2.7606(12) Å (Table 1). These Sr–N/O distances are within the normal range and are consistent with the corresponding chemical bond lengths in other Sr(II) complexes reported in the literature [44,45].

**Figure 2:**
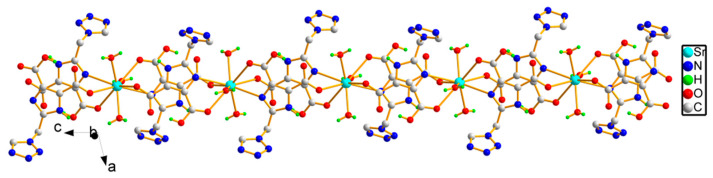
The infinite chain of **1** along the *c*-axis.

**Figure 3:**
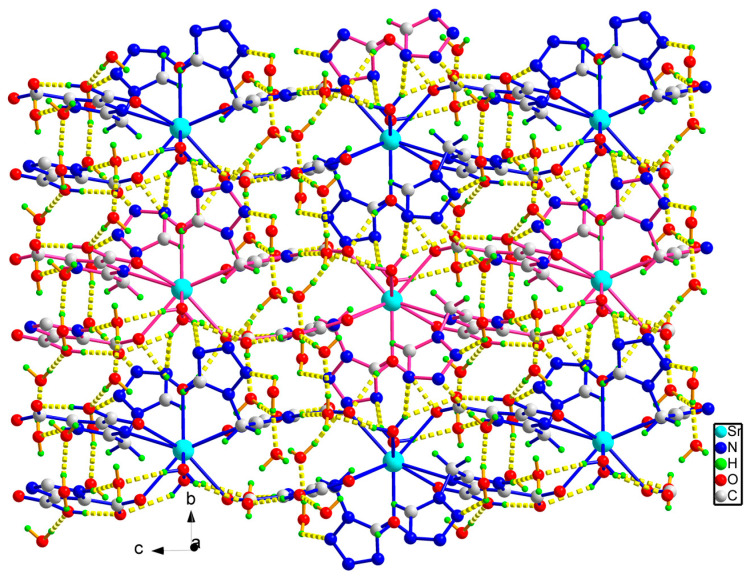
The two-dimensional sheet of **1** bearing H-bonds.

**Figure 4:**
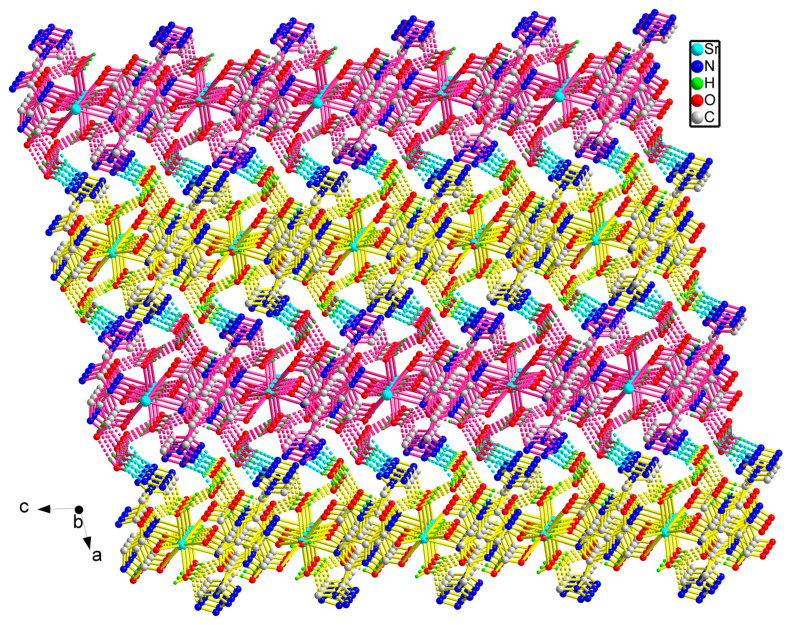
The three-dimensional solid-state framework of **1** (Blue: N; Red: O; Green: H; Gray: C; ultramarine: Sr).

The adjacent Sr(II) ions were joined to each other by the bridging ligand H_2_tmidc^−^ to form a long chain extending indefinitely along the *c*-direction, where the distance between adjacent Sr(II) ions is 8.8476(3) Å (Figure 2). In addition to the intramolecular O-H⋯O H-bonding link between carboxyl units, there are O-H⋯N H-bonds between coordination water and tetrazolium rings between chains, and O-H⋯O H-bonds between carboxyl units, crystalline water, and coordination H_2_O (please see Figure 3 for an array of these H-bonds). As a result, the chains are linked together by the seven kinds of hydrogen bonds mentioned above (Table 2), forming the two-dimensional layered structure (Figure 3). Figure 4 depicts a three-dimensional structure of **1** with layers joined by two kinds of O-H⋯N hydrogen-bonding connections. Furthermore, there is a significant π-π interaction between the neighboring imidazole units, and the distance between the centroids of the adjacent imidazole rings is 3.5776(7) Å. This adds stability to the three-dimensional solid structure.

To summarize, compound **1** has a large number of free tetrazolium groups, imidazole groups, carboxyl oxygen atoms, coordination, and crystalline H_2_O units. The complicated interaction between these groups will generate a dense and rich hydrogen bond network, ensuring the compound’s efficient proton transport.

### 2.2. Infrared Spectral Analysis

As denoted in Figure S1, the infrared spectra of organic ligand H_3_tmidc and its equivalent MOF **1** are comparable but not identical, as discussed below. For H_3_tmidc, the wide absorption band at 3540 cm^−1^ is attributed to the N-H stretching vibration. The absorption is NH in-plane bending vibration at 1477 cm^−1^ and C=N stretching vibration at 1551 cm^−1^. Continuous strong absorption peaks at 1476–1551 cm^−1^ manifest the presence of tetrazole rings. The absorption peaks at 1716 and 1476 cm^−1^ are ascribed to C=O stretching vibration and the in-plane bending of OH, respectively. The absorption peaks at 1369 and 1331 cm^−1^ are the stretching vibration of C-O. For **1**, compared with H_3_tmidc, the infrared characteristic peaks of ν_OH_ expansion vibration are blue-shifted and the absorption peaks are enhanced. That means an O-Sr coordination bond is formed. The blue shift of the absorption peak of ν_C=N_ indicates that N participates in the coordination. The above fully indicates that H_3_tmidc does indeed coordinate with the metal Sr(II).

### 2.3. Stability Analysis

To determine the thermal stability of **1** in an air atmosphere, a certain number of microcrystalline samples were precisely weighed to investigate the thermal degradation behavior. As depicted in Figure S2, the weight loss from 25 to 115 °C is due to the loss of four crystalline H_2_O units (found 10.71%, calculated 10.46%). As the temperature rises, three coordination water molecules are lost, which continues until 216 °C (found 7.66%, calculated 7.85%). As the temperature rose, the organic components within the framework continued to undergo intense thermal breakdown, which lasted until 795 °C. A constant weight platform then forms, indicating the creation of strontium oxide (found 14.88%, calculated 15.05%). The above test results reveal that, whereas the crystalline water molecules of compound **1** are lost below 100 °C, the coordinated water molecules remain, giving a material basis for the study of proton transport in water vapor.

**Figure 5:**
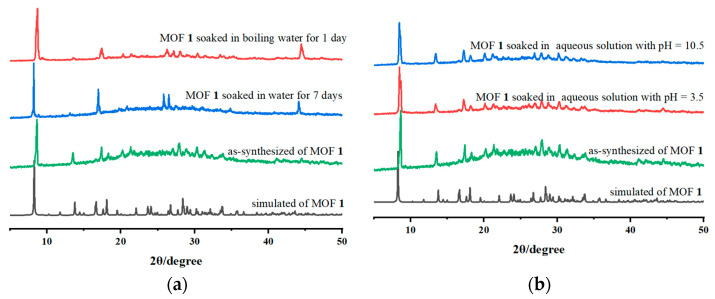
PXRD patterns of **1** before and after H_2_O treatment (**a**) or acidic and basic solutions treatment (**b**).

A high-quality proton-conductive material must be resistant to water and chemically inert, making it suitable for future practical applications. Consequently, we looked into compound **1**’s chemical and water stability in more detail. Initially, a suitable quantity of microcrystalline samples was immersed in water for either a week at room temperature or a day at boiling temperature. Subsequently, the collected samples were dried and used for PXRD tests to ascertain compound **1**’s H_2_O stability. As denoted in Figure 5a, the PXRD patterns of samples for **1** before and after H_2_O treatment are consistent with the simulated ones from crystal data, manifesting high water stability.

Moreover, to demonstrate the chemical stability of compound **1**, two suitable microcrystalline samples were submerged in acidic (pH = 3.5) or basic (pH = 10.5) aqueous solutions for 1 d. The samples were collected, washed with H_2_O, and dried for PXRD pattern testing and comparison. As unveiled in Figure 5b, the overlapped PXRD patterns demonstrated that **1** had good chemical stability.

### 2.4. Porosity Analysis

Under 77 K, the N_2_ adsorption/desorption isotherm was determined by employing the activated sample of MOF **1** (Figure S3). According to the IUPAC classification, this is a typical type III adsorption/desorption isotherm. As displayed in Figure S3, the N_2_ uptake boosts with the rising *P*/*P*_0_ and attains the highest value of 96.04 cm^3^/g STP as *P*/*P*_0_ = 0.99. At relative pressures ranging from 0.8 to 0.99, the adsorption and desorption curves exhibit a degree of variation indicating a specific interaction between the nitrogen molecules and the framework components. Its Brunauer–Emmett–Teller (BET) surface area was 27.0 m^2^/g, and the average Barret–Joyner–Halenda (BJH) pore size and pore volume were 18.9 nm and 0.15 cm^3^/g, respectively. Compound **1** may have a small aperture size with small volume and has no prominent pores based on the nitrogen adsorption/desorption results.

### 2.5. Proton-Conducting Properties

The AC impedance spectra of **1** at variable temperatures and humidity were measured using the quasi-four-electrode method. The compound’s proton conductivity (Table 3) was then assessed using the AC impedance test findings. Based on this, we investigated the relationship between σ and temperature and humidity and the mechanism of proton conduction.

**Figure 6:**
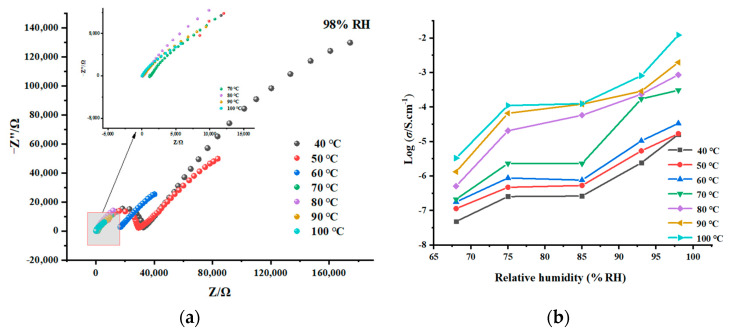
Nyquist spectra of **1** under 98% RH and different temperatures (**a**). Dependence diagram of σ for **1** and temperature at the fixed RHs (**b**).

First, by varying the temperature at a constant relative humidity, the relationship between compound **1**’s proton conductivity and temperature was examined. We set five humidity conditions (68%, 75%, 85%, 93% and 98% RHs) to test the effect of temperature on σ. The determined AC impedance spectra at a fixed RH and variable temperatures were exhibited in Figure 6a and Figures S4–S7, respectively. The Nyquist diagram for compound **1** at 98% RH, as shown in Figure 6a, has a slope in the low-frequency zone and a semi-arc in the high-frequency region. As temperature rises, the semicircle gets smaller and eventually turns into a full slope. This implies that the compound’s framework exhibits conventional proton conduction behavior [46,47,48,49], and the compound’s AC impedance lowers as temperature increases, increasing proton conductivity. Similarly, the same changes in the Nyquist plots with increasing temperature are visible at other relative humidity levels (Figures S4–S7). As listed in Table 3, the σ values vary from 1.62 × 10^−5^ (40 °C) to 1.22 × 10^−2^ S/cm (100 °C) under 98% RH [from 2.42 × 10^−6^ (40 °C) to 8.13 × 10^−4^ S/cm (100 °C) under 93% RH; from 2.64 × 10^−7^ (40 °C) to 1.26 × 10^−4^ S/cm (100 °C) under 85% RH; from 2.55 × 10^−7^ (40 °C) to 1.11 × 10^−4^ S/cm (100 °C) under 75% RH; from 7.84 × 10^−8^ (40 °C) to 3.02 × 10^−6^ S/cm (100 °C) under 68% RH]. It has been discovered that at each set humidity, the σ of **1** soars dramatically with temperature, with the difference between the lowest and greatest values being 2–3 orders of magnitude. Figure 6b illustrates the propensity of proton conductivity to grow fast as temperature rises. The following explanations can be used to explain compound **1**’s positive association between temperature and proton conductivity. First, as temperature rises, more energy can be absorbed by the coordination and crystalline water molecules in the framework or by water molecules adsorbed from the outside, which causes more hydrated protons to dissociate and increases the number of proton sources. Second, as temperature rises, more hydrated protons vibrate faster, which causes more orientational changes that are more favorable for the efficient transmission of protons and, ultimately, a sharp rise in proton conductivity.

**Figure 7:**
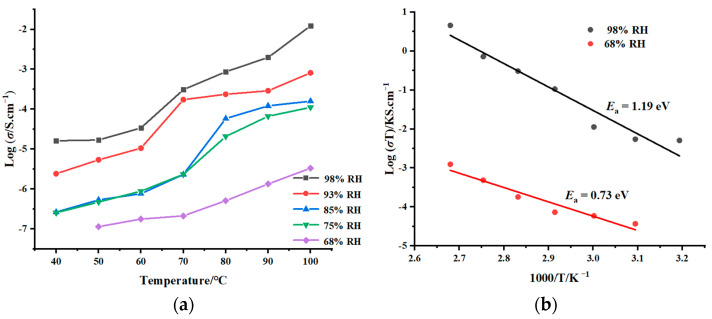
Dependence diagram of σ for **1** and RH at the fixed temperatures (**a**). The evaluated *E*_a_ values of **1** under 68% and 98% RHs (**b**).

Additionally, at a given temperature, the relationship between compound **1**’s proton conductivity and relative humidity was examined. As manifested in Figure 7a, from 40 to 100 °C, the σ values boost rapidly with the rising RHs. For instance, under 40 °C, the σ values are from 4.84 × 10^−4^ (68% RH) to 1.62 × 10^−5^ S/cm (98% RH). Under 100 °C, the σ values are from 3.02 × 10^−6^ (68% RH) to 1.22 × 10^−2^ S/cm (98% RH) (Table 3). Proton conductivity exhibits a rapid increase with increasing humidity, increasing by 3–4 orders of magnitude at different temperatures. This phenomenon suggests that an increase in the number of water molecules in the external environment can greatly promote the adsorption of more water molecules in the framework of **1**, increasing the number of proton sources in the pore and a denser hydrogen bond network in the framework, allowing for faster proton transfer. Note that although proton conductivity typically increases rapidly with increasing humidity, it increases slowly between 75% and 85% RHs or at specific temperatures. This could be owing to inadequate water molecules adsorbed by the framework at 75–85% RHs or temperature circumstances that prevent the complete completion of water molecular deprotonation.

Excitingly, compound **1** has an optimal σ of 1.22 × 10^−2^ S/cm under 98% RH/100 °C, which is comparable to the excellent proton conductivity of MOF materials reported in the literature [50,51,52] and far greater than the σ of some MOFs built by other N-heterocyclic carboxylic acid ligands [35,36,37,38,39], demonstrating the significant construction advantages of the ligands used in this paper.

To gain insight into the proton-conducting mechanism of MOF **1**, based on the measured proton conductivity data and the Arrhenius equation, we performed linear fitting, estimated the corresponding activation energy data (Figure 7b) at 98% and 68% RH, and then predicted the proton transport behavior within the framework using the crystal structure data. As denoted in Figure 7b, the assessed activation energy (*E*_a_) values under 98% and 68% RHs are 1.19 and 0.73 eV, showing that the *E*_a_ values of **1** are much greater than 0.4 eV at both high and low humidity, which indicates that proton transfer in the framework mainly obeys the vehicular mechanism [8,53]. The reason for this phenomenon could be that the existence of a great number of strong intramolecular H-bonds in the framework of compound **1** weakens the construction of the continuous dense intermolecular hydrogen bond network, preventing protons from completing the “jump” through the hydrogen bond network, which can only be completed via hydrated proton-directed migration. Nonetheless, based on our examination of crystal structure and hydrogen bond data, we conclude that the proton transfer process in the framework is inextricably linked to the hydrogen bond network, implying that the Grotthuss mechanism is accompanied.

## 3. Materials and Methods

### 3.1. Materials

All reagents purchased from Sinopharm Group or Macklin Reagent Co., Ltd.(Shanghai, China) are of analytically pure grade unless otherwise specified without further purification. Organic ligand H_3_tmidc was prepared according to the reference method [54]. Strontium chloride hexahydrate (SrCl_2_·6H_2_O, 99.9%), methyl alcohol (MeOH, 99.99%), hydrochloric acid (HCl, 36–38%), sodium hydroxide (NaOH, 96.0%), and deionized H_2_O were used.

### 3.2. Determinations

The infrared spectrum (KBr, 400–4000 cm^−1^) was obtained by a Nicolet NEXUS 470-FTIR analyzer (Kyoto, Japan). PXRD plots could be obtained on a Rigaku D/MAX-3 diffractometer (Cu target; λ = 1.5418 Å) (Rigaku Corporation, Akishima, Japan). TGA was carried out on a NETZSCH STA 409PC analyzer (NETZSCH Corporation, Selb, Germany) [air flow; 10 °C/min]. Nitrogen gas (77 K) adsorption/desorption isothermal was obtained from the ASAP 2420 instrument (Micromeritics Corporation, Norcross, GA, USA).

### 3.3. Preparation of MOF 1

A quantity of 0.06 mmol H_3_tmidc (0.0143 g) was dissolved in 2 mL methanol (called liquid A), and 0.06 mmol SrCl_2_·6H_2_O (0.0160 g) was dissolved in 2 mL deionized water (called liquid B). Then, liquid A is added to liquid B drop by drop and mixed evenly. The clarified solution is filtered and left at 25 °C for 10 days. Light yellow needle-like transparent crystals for **1** were obtained, washed with H_2_O, and dried naturally in air. The yield was 54.2%, based on the H_3_tmidc. Anal. calculated for C_14_H_24_N_12_O_15_Sr: C, 24.43; H, 3.52; N, 24.44%. Found: C, 24.30; H, 3.08; N, 24.61%. IR (cm^−1^, KBr): 3462 (br), 3147 (w), 3027 (w), 1697 (m), 1621 (m), 1599 (w), 1541 (s), 1424 (w), 1373 (m), 1245 (w), 1178 (w), 1111 (m), 1031 (m), 976 (w), 906 (m), 780 (m), 671 (m), 656(w), 502 (m), 418 (w).

### 3.4. Crystal Structure Measurement

A light yellow high-quality single crystal of compound **1** with a size of 0.22 × 0.21 × 0.16 mm^3^ is glued to a thin glass fiber. A molybdenum target microfocal source (λ = 0.71073 A) was tested on a Bruker D8 VENTURE PHOTON double microfocal X-ray single crystal diffractometer at T = 300(2) K. Data were collected in the range of 2.709–27.505 degrees using ω and φ scanning. SHELXT software (v6.2) package was used to obtain the initial model of the crystal structure by a direct approach, and then the SHELXL crystallography software package [55,56] was used to refine the coordinates of all non-hydrogen atoms by the total matrix least squares method based on F^2^. The H on C and N is obtained via theoretical hydrogenation, and the hydrogen on H_2_O is measured in the light of the residual peak and the direction of the H bond. All non-H atoms undergo anisotropic refinement, while hydrogen atoms undergo isotropic refinement. The crystallographic parameters of **1** are listed in Table 4. CCDC no. is 2353666.

### 3.5. Stability Determinations

The general procedure for testing the water and chemical stability of compound **1** is as follows: weigh the appropriate weight of the microcrystalline sample of **1**, soak it in room temperature or boiling water for seven days or one day, respectively, or soak it in acidic (HCl) or alkaline (NaOH) aqueous solutions for one day, then centrifuge the solids, dry them in the air, and perform PXRD analysis.

### 3.6. Proton-Conducting Determinations

The Princeton 2273 electrochemical Station was used in a quasi-four-electrode manner to test compound **1**’s AC impedance profile (AC voltage: 100 mV; 1–10^6^ Hz).

Because it is hard to obtain a single-crystal sample of a suitable size, only microcrystal samples of **1** can be used for electrochemical testing via the pressing tablet method. The disc sample was manufactured as described previously [36,37]: approximately 35 mg of the microcrystalline sample was placed into a disc mold and allowed to rest for 3 min at a pressure of 3.5 Mpa, and then the disc was collected. Vernier caliper was used to precisely measure the radius and thickness. Then, it was sandwiched between two silver electrodes, suspended in a humid environment, and balanced for 18 h, and the AC impedance map was recorded. A constant temperature and humidity box (Shanghai YiHeng BPS-50CL (Shanghai YiHeng Co. LTD. Shanghai, China)) regulates the 68–98% RHs and 30–100 °C levels.

The following two equations were used to calculate the σ and *E*_a_: σ = L/(RS) and Tσ = σ_0_exp(−*E*_a_/kT), respectively [50,51,52].

### 3.7. The Simulation of Nyquist Plots

The equivalent circuit LR(OR)(CR) of **1** was achieved by employing ZSimpWin (v3.6) to simulate the Nyquist spectra under 30/100 °C and 98% RH (Figure S8).

## 4. Conclusions

This study reports the successful preparation of a one-dimensional chain strontium(II)-based MOF with good thermal stability, water stability, and chemical inertia utilizing a multi-functional organic bridge ligand of dicarboxylic acid comprising tetrazolium and imidazole groups. These lengthy chains produce a three-dimensional solid- structure with a dense hydrogen bond network as a result of intermolecular and intramolecular hydrogen bonding and π-π packing force. The compound demonstrated excellent proton conductivity with the best value of 10^−2^ S/cm, which was significantly higher than the σ of MOFs built by imidazole dicarboxylates reported in the literature. It was also ranked at the forefront of excellent proton-conductive MOFs, indicating potential applications in fuel cells and other electrochemical fields. The design and construction of high-performance proton-conductive crystalline materials have new opportunities as a result of this work.

## Figures and Tables

**Table 1:** Important bond distances (Å) and bond angles (°) of **1**.

Sr(1)-O(6)	2.545(2)	Sr(1)-O(5)	2.5634(14)
Sr(1)-O(5)#1	2.5634(14)	Sr(1)-O(1)#1	2.6712(12)
Sr(1)-O(1)	2.6713(12)	Sr(1)-O(4)#2	2.7606(12)
Sr(1)-O(4)#3	2.7606(12)	Sr(1)-N(1)#1	2.8083(12)
Sr(1)-N(1)	2.8084(12)		
O(6)-Sr(1)-O(5)	113.95(4)	O(6)-Sr(1)-O(5)#1	113.95(4)
O(5)-Sr(1)-O(5)#1	132.09(8)	O(6)-Sr(1)-O(1)#1	69.00(3)
O(5)-Sr(1)-O(1)#1	135.51(4)	O(5)#1-Sr(1)-O(1)#1	65.02(4)
O(6)-Sr(1)-O(1)	69.00(3)	O(5)-Sr(1)-O(1)	65.02(4)
O(5)#1-Sr(1)-O(1)	135.51(4)	O(1)#1-Sr(1)-O(1)	138.00(6)
O(6)-Sr(1)-O(4)#2	138.55(2)	O(5)-Sr(1)-O(4)#2	71.62(4)
O(5)#1-Sr(1)-O(4)#2	72.94(5)	O(1)#1-Sr(1)-O(4)#2	137.49(4)
O(5)-Sr(1)-O(4)#3	72.94(5)	O(5)#1-Sr(1)-O(4)#3	71.62(4)
O(1)#1-Sr(1)-O(4)#3	78.46(4)	O(1)-Sr(1)-O(4)#3	137.50(4)
O(4)#2-Sr(1)-O(4)#3	82.90(5)	O(6)-Sr(1)-N(1)#1	71.68(2)
O(5)-Sr(1)-N(1)#1	77.50(4)	O(5)#1-Sr(1)-N(1)#1	118.14(4)
O(4)#2-Sr(1)-N(1)#1	143.88(4)	O(4)#3-Sr(1)-N(1)#1	70.32(3)
O(6)-Sr(1)-N(1)	71.68(3)	O(5)-Sr(1)-N(1)	118.14(4)
O(1)-Sr(1)-N(1)	60.93(3)	O(4)#2-Sr(1)-N(1)	70.32(3)
O(4)#3-Sr(1)-N(1)	143.88(3)	N(1)#1-Sr(1)-N(1)	143.37(5)

Symmetry modes: #1: −x + 2, y, −z + 3/2; #2: −x + 2, −y + 3, −z + 2; #3: x, −y + 3, z − 1/2.

**Table 2:** H-bonding parameters of **1**.

D-H⋯A	*d*(D-H) (Å)	*d*(H⋯A) (Å)	*d*(D⋯A) (Å)	(D-H⋯A)(°)
N(2)-H(2)⋯O(7)#4	0.86	1.96	2.8100(18)	167.3
O(2)-H(2A)⋯O(3)	0.82	1.64	2.4596(17)	176.4
O(5)-H(5A)⋯O(7)#5	0.85	2.11	2.961(2)	173.9
O(5)-H(5B)⋯N(6)#6	0.81	2.50	3.073(2)	128.2
O(5)-H(5B)⋯O(3)#3	0.81	2.36	3.0559(19)	144.7
O(6)-H(6C)⋯O(4)#7	0.85	1.98	2.8050(19)	162.6
C(6)-H(6A)⋯O(8)#8	0.97	2.47	3.374(2)	155.1
O(7)-H(7A)⋯O(8)	0.85	1.95	2.794(2)	173.1
O(7)-H(7B)⋯N(4)#9	0.85	2.51	3.121(2)	129.4
O(8)-H(8A)⋯O(2)#10	0.85	2.06	2.8959(19)	169.6
O(8)-H(8B)⋯N(5)	0.85	2.31	3.144(3)	167.5

Symmetry modes: #3: *x*, −*y* + 3, *z* − 1/2; #4: −*x* + 3/2, −*y* + 3/2, −*z* + 2; #5: *x* + 1/2, *y* + 3/2, *z*; #6: −*x* + 2, *y* + 1, −*z* + 3/2; #7: −*x* + 2, −*y* + 2, −*z* + 2; #8: *x*, *y* + 1, *z*; #9: *x*, *y* − 1, *z*; #10: *x* − 1/2, *y* − 1/2, *z*.

**Table 3:** The σ values (S/cm) of **1** under variable temperatures and RHs.

T (°C)	68% RH	75% RH	85% RH	93% RH	98% RH
40	4.84 × 10^−8^	2.55 × 10^−7^	2.64 × 10^−7^	2.42 × 10^−6^	1.62 × 10^−5^
50	1.14 × 10^−7^	4.73 × 10^−7^	5.34 × 10^−7^	5.38 × 10^−6^	1.69 × 10^−5^
60	1.77 × 10^−7^	8.85 × 10^−7^	7.66 × 10^−7^	1.05 × 10^−5^	3.39 × 10^−5^
70	2.12 × 10^−7^	2.33 × 10^−6^	2.31 × 10^−6^	1.73 × 10^−4^	3.08 × 10^−4^
80	5.12 × 10^−7^	2.07 × 10^−5^	5.81 × 10^−5^	2.37 × 10^−4^	8.66 × 10^−4^
90	1.34 × 10^−6^	6.64 × 10^−5^	1.21 × 10^−4^	2.90 × 10^−4^	1.98 × 10^−3^
100	3.02 × 10^−6^	1.11 × 10^−4^	1.26 × 10^−4^	8.13 × 10^−4^	1.22 × 10^−2^

**Table 4:** The crystallographic parameters of **1**.

Compound	1
Empirical formula	C_14_H_24_N_12_O_15_Sr
Formula weight	688.07
Temperature, K	300(2) K
Crystal size, mm^3^	0.22 × 0.21 × 0.16
Crystal system	Monoclinic
Space group	C2/c
*a*, Å	21.8700(10)
*b*, Å	6.7421(2)
*c*, Å	17.6471(7)
*α*, deg	90
*β*, deg	101.865(2)
*γ*, deg	90
Volume, Å^3^	2546.47(17)
Z	4
Calculated density, g cm^−3^	1.795
Absorption coefficient, mm^−1^	2.215
*F*(000), e	1400
*θ* range for data collection, deg	2.709–27.505
Radiation	Mo K*α*
Index ranges	−28 ≤ h ≤ 2 8, −8 ≤ k ≤ 8, −22 ≤ l ≤ 22
Reflections collected/unique	59,724/2920
*R*_int_	0.0477
Data/restraints/parameters	2920/0/192
Final indices *R*_1_/*wR*_2_ [*I* > 2*σ*(*I*)]	0.0228/0.0594
Final indices *R*_1_/*wR*_2_ (all data)	0.0255/0.0608
Goodness-of-fit on (*F*^2^)	1.030
Δ*ρ*_fin_ (max/min), e·Å^−3^	0.495/−0.333

## Data Availability

Data are contained within the article.

## Supplementary Materials

The following supporting information can be downloaded at: https://www.mdpi.com/article/10.3390/molecules29112656/s1, Figure S1: IR spectra of ligand H_3_tmidc (a) and MOF **1**(b); Figure S2: TG carve of MOF **1**; Figure S3: The N_2_ adsorption/desorption isotherm of MOF **1**; Figure S4: Nyquist spectra of **1** under 93% RH and different temperatures; Figure S5: Nyquist spectra of **1** under 85% RH and different temperatures; Figure S6: Nyquist spectra of **1** under 75% RH and different temperatures; Figure S7: Nyquist spectra of **1** under 68% RH and different temperatures; Figure S8: Nyquist plots for **1** at 30 (a) or 100 °C (b) and 98% RH. Red circle and green square are the measured impedance spectroscopy values and the fits of the impedance data to the equivalent circuit of LR(OR)(CR).
