# Supplementary material for: High H2O-Assisted Proton Conduction in One Highly Stable Sr(II)-Organic Framework Constructed by Tetrazole-Based Imidazole Dicarboxylic Acid
Source: Molecules. 2024 Jun 4;29(11):2656. doi: 10.3390/molecules29112656 (PMC11173819; doi:10.3390/molecules29112656)
Supplement: Supplementary file 1 [file molecules-29-02656-s001.zip › molecules-3044479-supplementary.pdf]

Supporting information

High H_2O-Assisted Proton Conduction in One Highly Stable Sr(II)-Organic Framework Constructed by Tetrazole-Based Imidazole Dicarboxylic Acid

Junyang Feng [1], Ying Li [1], Lixia Xie [2], Jinzhao Tong [3] and Gang Li [3,*]

[1] School of Pharmaceutical Engineering, Henan Technical Institute, Zhengzhou 450042, China; feng329426@sina.com (J.F.); yechenmo1989@126.com (Y.L.)
[2] College of Science, Henan Agricultural University, Zhengzhou 450002, China; henauxlx@henau.edu.cn
[3] College of Chemistry, Zhengzhou University, Zhengzhou 450001, China; tongjch2023@lzu.edu.cn
* Correspondence: gangli@zzu.edu.cn

Number of pages: 6
Number of Figures: 8

List

Figure S1. IR spectra of ligand H_3tmidc (a) and MOF **1**(b);
Figure S2. TG carve of MOF 1;
Figure S3. The N_2 adsorption/desorption isotherm of MOF **1**;
Figure S4. Nyquist spectra of **1** under 93% RH and different temperatures;
Figure S5. Nyquist spectra of **1** under 85% RH and different temperatures;
Figure S6. Nyquist spectra of **1** under 75% RH and different temperatures;
Figure S7. Nyquist spectra of **1** under 68% RH and different temperatures;
Figure S8. Nyquist plots for **1** at 30 (a) or 100 °C (b) and 98% RH. Red circle and green square are the measured impedance spectroscopy values and the fits of the impedance data to the equivalent circuit of LR(OR)(CR).

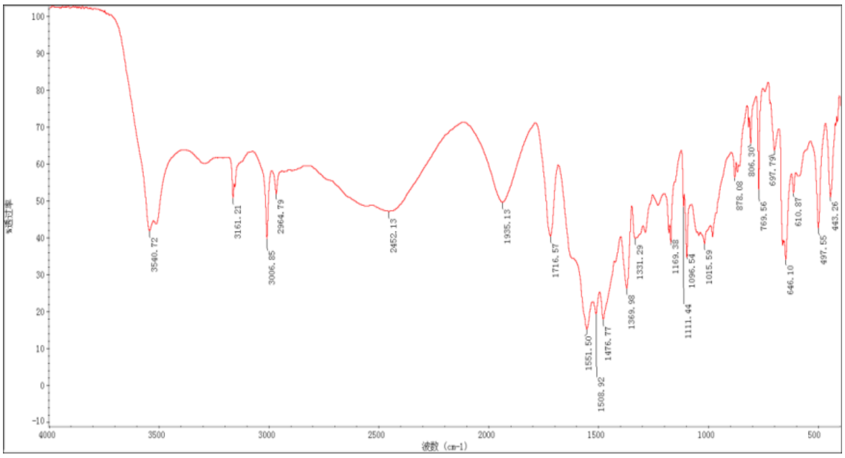

(a)

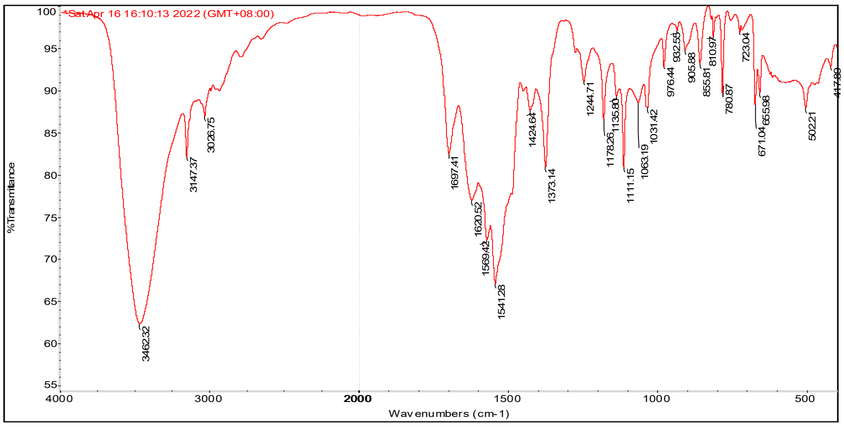

(b)

Figure S1. IR spectra of ligand H_3tmidc (a) and MOF **1**(b).

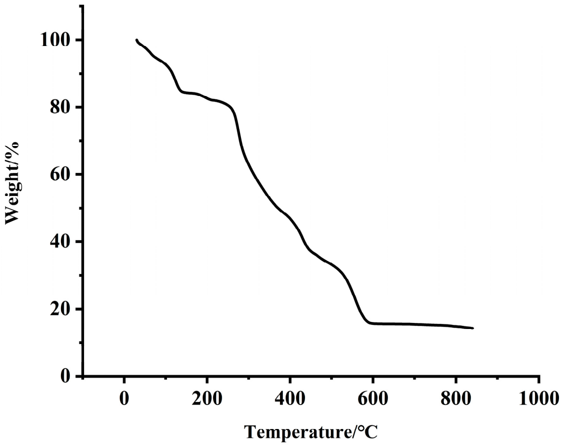

Figure S2. TG carve of MOF **1**.

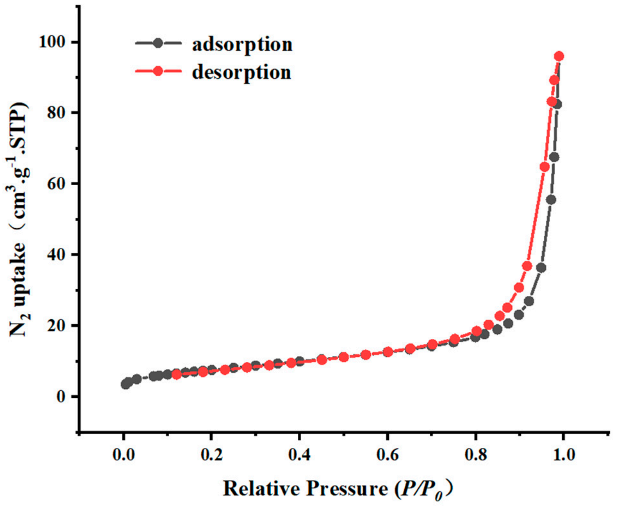

Figure S3. The N_2 adsorption/desorption isotherm of MOF **1**.

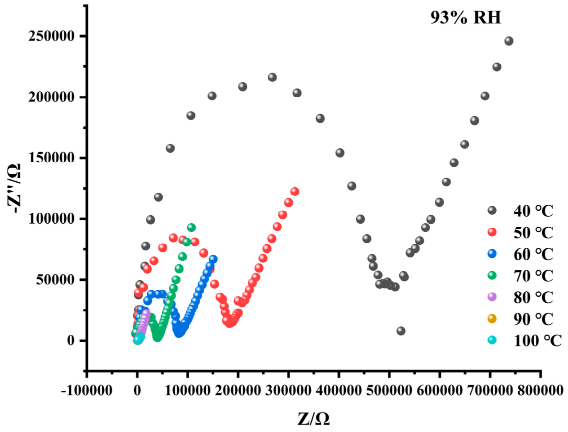

Figure S4. Nyquist spectra of **1** under 93% RH and different temperatures.

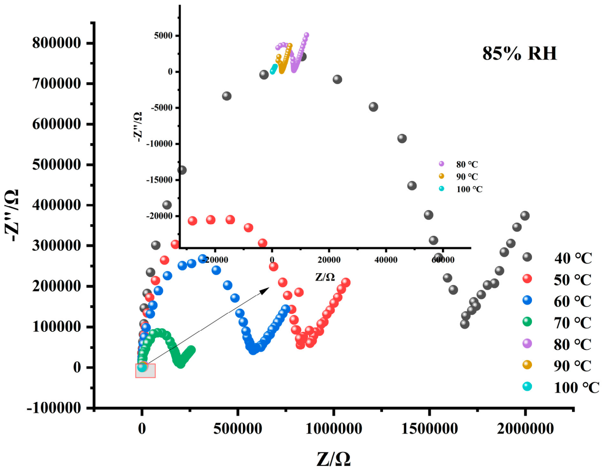

Figure S5. Nyquist spectra of **1** under 85% RH and different temperatures.

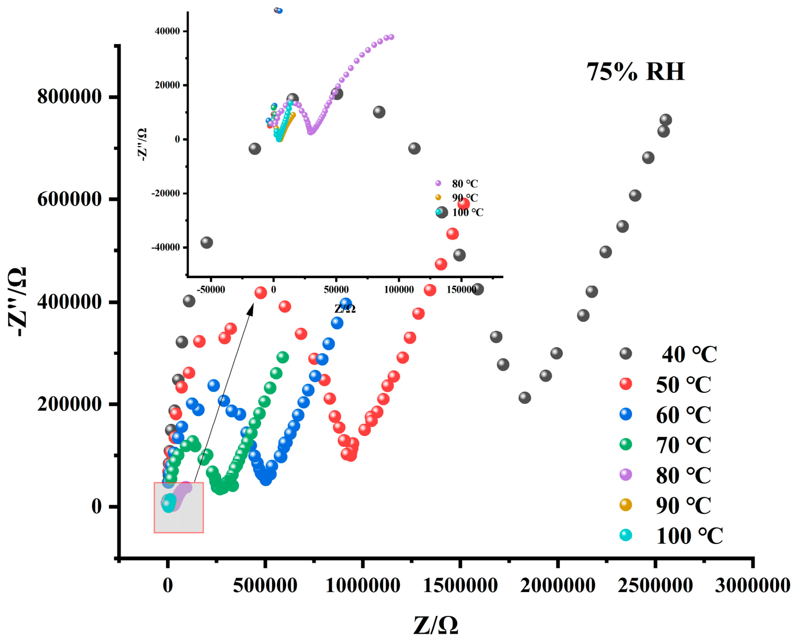

Figure S6. Nyquist spectra of **1** under 75% RH and different temperatures.

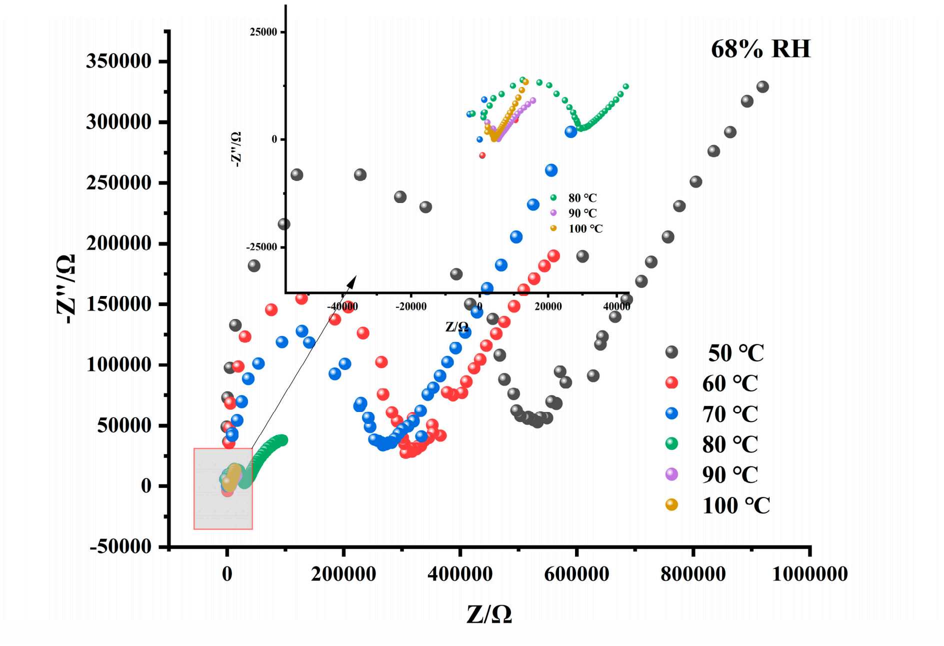

Figure S7. Nyquist spectra of **1** under 68% RH and different temperatures.

The equivalent circuit diagrams for **1** were obtained by simulating the AC impedance data at 30 °C and 100 °C at 98% RH using ZSimpWin software.

Equivalent circuit LR(OR)(CR):

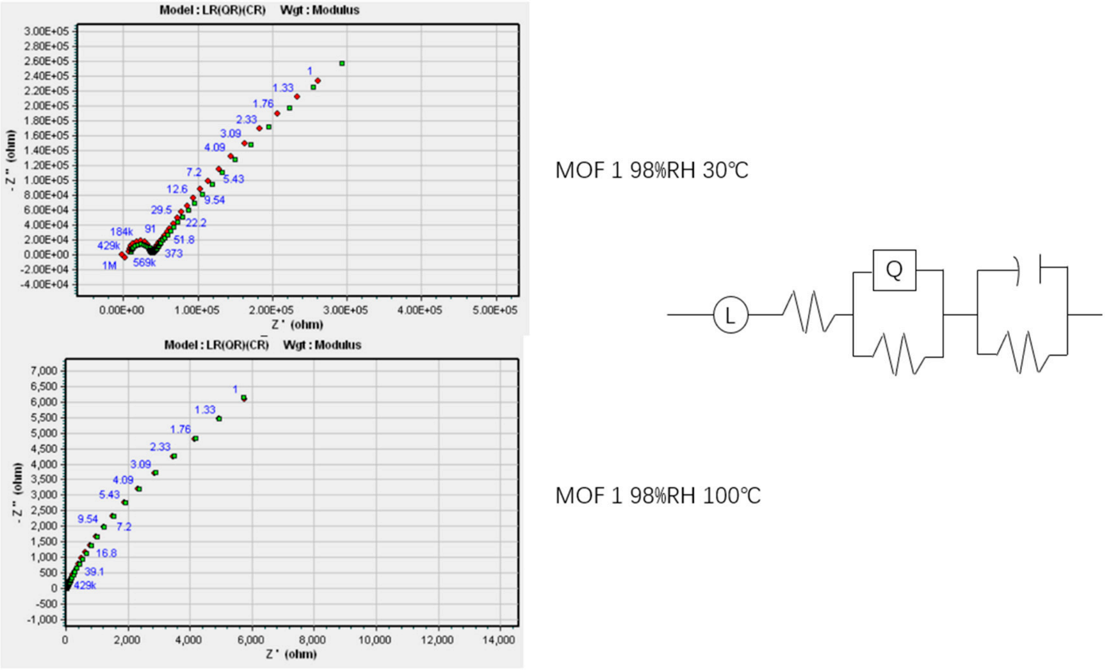

Figure S8. Nyquist plots for **1** at 30 (a) or 100 °C (b) and 98% RH. Red circle and green square are the measured impedance spectroscopy values and the fits of the impedance data to the equivalent circuit of LR(OR)(CR).
